# Molecular and isotopic evidence for the processing of starchy plants in Early Neolithic pottery from China

**DOI:** 10.1038/s41598-018-35227-4

**Published:** 2018-11-19

**Authors:** Shinya Shoda, Alexandre Lucquin, Chi Ian Sou, Yastami Nishida, Guoping Sun, Hiroshi Kitano, Joon-ho Son, Shinichi Nakamura, Oliver E. Craig

**Affiliations:** 1 BioArCh, University of York, Wentworth Way, Heslington York, YO10 5NG UK; 2 Nara National Research Institute for Cultural Properties, Nijo 2-9-1, Nara, Nara, 630-8577 Japan; 3 Niigata Prefectural Museum of History, Sekihara 1, Nagaoka, Niigata 940-2035 Japan; 4 Zhejiang Provincial Institute of Relics and Archaeology, 26 Jiashan Xincun, Juashan Road, Hangzhou, Zhejiang 310014 China; 5 Tohoku University of Art and Design, Kamisakurada 3-4-5, Yamagata, Yamagata, 990-9530 Japan; 6 Korea University, 2511 Sechong-ro, Jochiweon-up, Sejong-si 339-700 South Korea; 7 Kanazawa University, Kakuma, Kanazawa, Ishikawa, Ishikawa, 920-1192 Japan

## Abstract

Organic residue analysis of ancient ceramic vessels enables the investigation of natural resources that were used in daily cooking practices in different part of the world. Despite many methodological advances, the utilization of plants in pottery has been difficult to demonstrate chemically, hindering the study of their role in ancient society, a topic that is especially important to understanding early agricultural practices at the start of the Neolithic period. Here, we present the first lipid residue study on the Chinese Neolithic pottery dated to 5.0 k - 4.7 k cal BC from the Tianluoshan site, Zhejiang province, a key site with early evidence for rice domestication. Through the identification of novel molecular biomarkers and extensive stable isotope analysis, we suggest that the pottery in Tianluoshan were largely used for processing starchy plant foods. These results not only highlight the significance of starchy plants in Neolithic southern China but also show a clear difference with other contemporary sites in northern Eurasia, where pottery is clearly orientated to aquatic resource exploitation. These differences may be linked with the early development of rice agriculture in China compared to its much later adoption in adjacent northerly regions.

## Introduction

Plants have played an important role in shaping human history that extends back well beyond their domestication to our more distant forager ancestors^1,2^. However, methodologies for evaluating how plants were utilized in the past and their relationship to prehistoric material culture is still developing and has been largely restricted to the microscopic identification of plant remains adhering to the surfaces of lithic tools or grinding stones^3–5^ and occasionally on pottery^6^. An alternative and extremely promising avenue of research has been the chemical determination of organic residues associated with artefacts. This approach has been widely deployed to identify the original contents of prehistoric pottery vessels used for cooking, storage and serving foods and in the production of other natural materials, such as waxes and adhesives. For the former, organic residue analysis has mainly focused on the identification of fatty animal products, oil rich seeds, and plant and insect waxes through the isotopic and molecular characterisation of preserved lipids which are particularly rich in these products^7–9^. Conversely, the identification of more starchy plant foods has been much harder to demonstrate with the suspicion, backed in part by experimental evidence^10^, that they leave little or no trace during cooking. This is unfortunate given that elucidating the early role and eventual dependence on starchy crops is a key theme in world prehistory^11^.

It is unlikely that starchy plant food, such as cereals, were simply not used in pottery. Agriculture and pottery frequently co-occurred and both were key elements of many early farming societies^12^. Similarly, starchy wild tubers and nuts were likely to have been important to pottery producing hunter-gatherers. Indeed the need to detoxify starch rich plants has been cited as a motivation for the invention of pottery and it would also seem logical that starchy foods would have needed to be processed prior to consumption, particularly to aid digestion^13^. Linking the utilization of cultivated grains directly with one of the most common forms of material culture recovered in archaeological sites (i.e. pottery) would have immense benefits for examining the dispersal of important crops and for examining their early culinary significance.

To date, the evidence for processing starchy plants in pottery is extremely sparse, with the most promising studies relating to the identification of lipid biomarkers for millet^14^, and maize^15^. Both of these are C_4_ crops and therefore have relatively enriched carbon isotope ratios compared to wild C3 plants, permitting further distinction. It has also been shown that early Holocene pottery from the Libyan Sahara was used by hunter-gatherers for processing plant materials by the identification of lipids consistent with seed oils from local wild plants^16^. Yet the routine identification of the most important domesticated C_3_ cereal crops, such as rice, wheat and barley in pottery, has been much more challenging. Recently, biomarkers for wheat and barley (alkylresorcinols) have been identified in Roman vessels buried in anoxic conditions^17^ and an exceptionally well preserved Early Bronze Age wooden container from an Alpine glacier^18^ but it is not known whether these compounds survive routinely in archaeological pottery. Similarly it is unclear whether extensive processing of wild starchy plants such as acorns or chestnuts would be detectable in pottery residues, although diagnostic compounds have been identified in charred deposits associated with storage pits from Japanese hunter-gatherer contexts^19^.

Here, we investigate the potential for identification of starchy plants at the Tianluoshan site in the Lower Yangtze river basin in Zhejiang Province, China; a waterlogged site that has preserved a remarkable assemblage of plant remains, including rice (*Oryza sativa*), foxnuts (*Euryale ferox*), water chestnuts (*Trapa spp*.) and acorns (*Lithocarpus spp*. and *Cyclobalanopsis spp*.). The site is particularly significant as the sequence captures the early stages of rice domestication, ca. 5,000 - 4,000 cal BC as this crop gradually replaced wild foraged foods to become an important staple^20^. However, the role that pottery played in this transition is unclear. Pottery production in China, as in most other regions of East Asia, predates agriculture by several thousand years with the earliest assemblages dating to the Late Glacial Maximum^21^ in the South. Indeed, analysis of hunter-gatherer pottery from Japan (Jomon) and Korea (Chulmun), shows a clear preference for processing of aquatic foods and surprisingly little evidence for plant processing despite the widespread availability of wild plant resources^22–25^.

In Japan there seems to be little change in the use of pottery at the start of the Holocene when the scale of production dramatically increases, and forest products are more abundant^23,25^. Even at later Jomon sites, with abundant evidence for intensive plant processing, pottery seems to have been used for processing fish^19^. However, in Japan there is no suggestion of cereal agriculture until much later at the Jomon-Yayoi cultural transition (ca. 800 cal BC). In China the situation is quite different as rice and millet are claimed to have been domesticated at the beginning of Holocene^26,27^ concomitant with increases in the scale of pottery production. Our study therefore provides an opportunity to investigate whether Holocene pottery in China followed a similar trajectory to other regions of East Asia or whether pottery was associated with the processing of wild and domesticated plants (i.e. rice).

**Figure 1:**
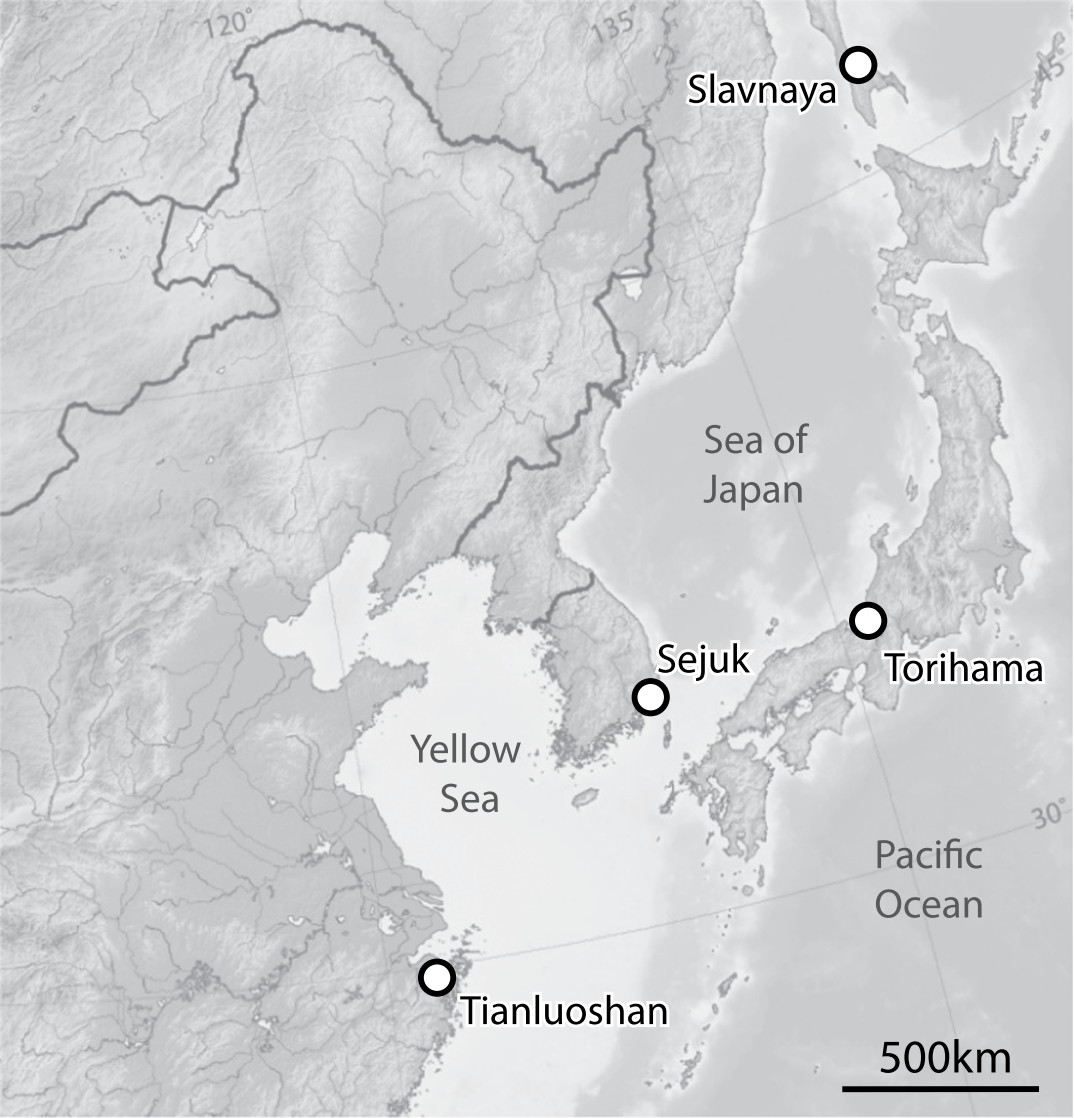
Map showing the location of Tianluoshan and other East Asian Early Holocene sites referred to in the text.

To tackle this question, organic residue analysis was undertaken on pottery from the early phases of the Tianluoshan archaeological site (30°01′N 121°22′E, Fig. 1) dating to Early Holocene (5.2 k to 4.7 k calBC, Table S1). The potsherds were stored in the on-site museum of Tianluoshan, after they were excavated (2010–2013) from the cultural layers. Ceramic powder samples were taken from upper body or rim of *Fu* type boiler (rim diameter; 17.9–33.9 cm), while adhering charred surface deposit (hereafter foodcrusts) samples were taken from the interior wall of the upper and bottom sherds of pottery. The ceramic vessels were excavated from the cultural layers along with densely spaced wooden structures, indicative of a settlement, most likely representing therefore domestic pottery use.

## Results

We conducted three independent lines of analysis to investigate pottery use at Tianluoshan. Firstly, carbon and nitrogen isotopes were measured in foodcrusts to broadly distinguish marine/freshwater/terrestrial sources^28^. Second, lipids extracted from both foodcrusts and from the ceramic matrices of the vessels were analysed by gas chromatography mass spectrometry to identify their likely source. Thirdly, carbon stable isotope values of individual lipids were measured to determine their origins by comparing with modern and archaeological authentic reference samples.

### Bulk isotope analysis

**Figure 2:**
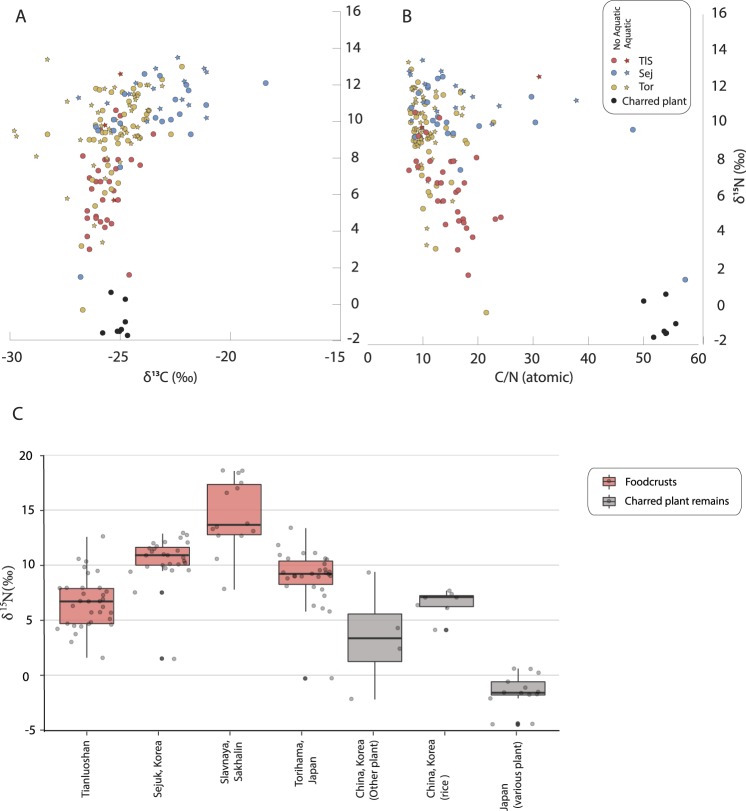
Bulk isotope characteristics of charred deposits on pottery (foodcrusts) and charred plant remains in East Asia. (**A**) Plot of δ^13^C and δ^15^N values obtained from foodcrusts from Tianluoshan (Tls, this study), Sejuk (Sej)^24^, Torihama (Tor)^23^ and charred plant remains from Sannai-Maruyama^19^. (**B**) Plot of carbon to nitrogen ratios and δ^15^N. (**C**) Boxplots comparing foodcrusts δ^15^N from Tianluoshan, Sejuk, Slavnaya (Sakalin)^29^ and charred rice grains and hazelnuts from Songdam-ri, Korea (this study), as well as rice remains, persimmon seed and acorn from Tianluoshan^30^.

Carbon and nitrogen stable isotope ratio was measured by elemental analysis isotope ratio mass spectrometry (EA-IRMS). The range of δ^15^N values in the foodcrusts from Tianluoshan (1.6‰ to 12.6‰; median 6.7‰) are significantly lower (Kruskal-Wallis H = 55.623, p < 0.5) than those from other early Holocene hunter-gatherer pottery from East Asia, such as Japan^22,23,25^, Korea^24^ and the Sakhalin Islands^29^ (median 9.2‰, 10.9‰ and 13.7‰ respectively, Fig. 2, Table S2). δ^15^N values broadly reflect an organism’s trophic position thus lower values are more indicative of protein from terrestrial sources compared to aquatic. At the comparator sites with relatively higher δ^15^N compared to Tianluoshan, it is clear that residues are derived from charred aquatic foods as lipid biomarkers for heated marine and freshwater oils were detected in many of these samples^23,24,29^. Instead, the Tianluoshan foodcrusts are comparable with values obtained from charred plant remains, including rice from this region. For example, the δ^15^N values of archaeological rice remains from Tianluoshan itself ^30^ and Songdamri sites in Korea^31^ range from 4.1–7.7‰. However, as other charred plant materials and potentially residues from terrestrial animals also overlap with this range, it is impossible to distinguish a plant input using this approach alone. The isotope values are in general higher than charred plant remains from the middle/late Jomon pre-agricultural site of Sannai Maruyama in Japan which are thought to be derived from wild forest products, such as acorns and chestnuts^19^.

### Gas chromatography Mass Spectrometry (GC-MS)

**Figure 3:**
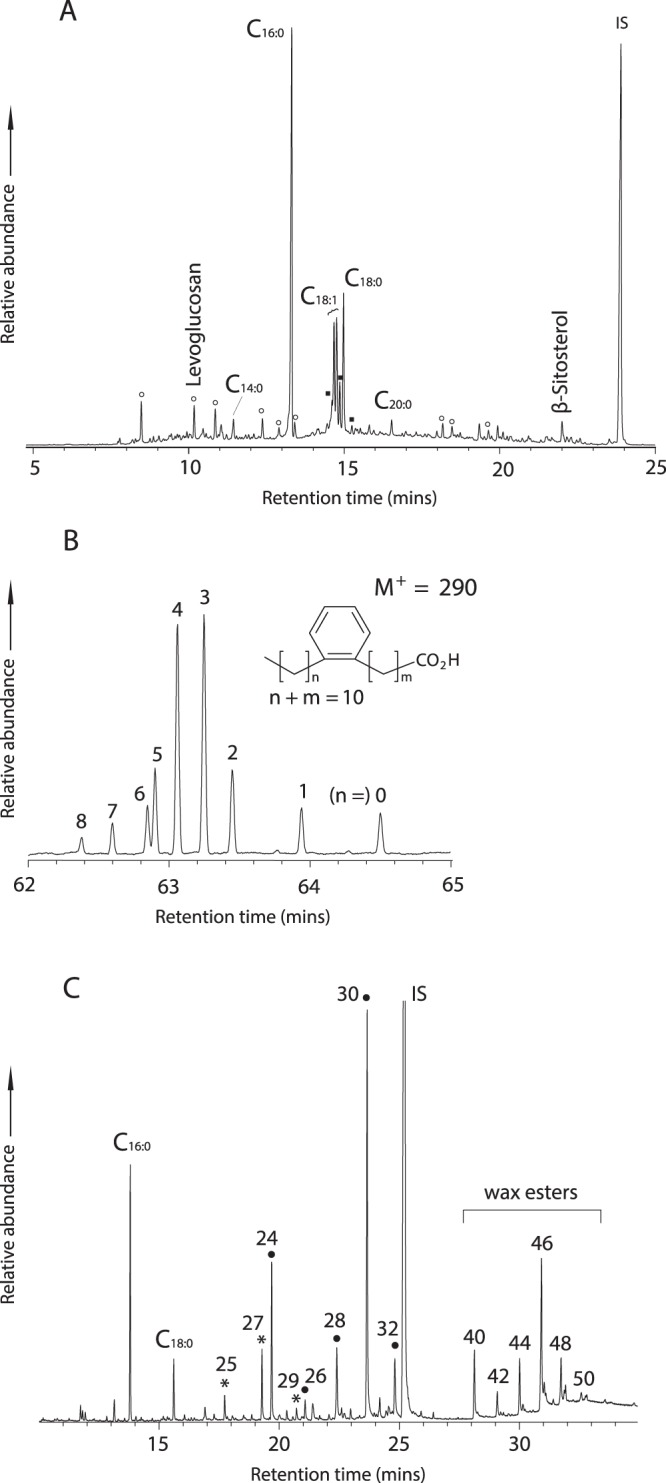
GC-MS analysis of Tianluoshan pottery. (**A**) Partial total ion chromatogram of a TLE from typical foodcrust associate with the Tianluoshan pottery vessels (TLS1016F) obtained by GC-MS using a DB1-HT column. Cx:y refers to fatty acids with x carbon atoms and y unsaturations. Filled squares are isomers of ω-(*o*-alkylphenyl)octadecanoic acid that partially co-elute with C_18:0_ and C_18:1_ fatty acids. Open circles are TMS derivatives of furanose and pyranoses sugars. (**B**) Mass chromatogram (*m/z* 290) showing isomeric distribution of C_18_ APAAs extracted with acidified methanol from a typical foodcrust (TLS035F) and resolved on a DB-23 column in SIM mode. (**C**) Partial total ion chromatogram of TLE from the Tianluoshan pottery foodcrust (TLS1015F) showing the existence of beeswax^38^. Asterisk - odd number alkanes with carbon chain length indicated; filled circles = alkanols with carbon chain length indicated. Cx:y means saturated fatty acids with x carbon length and number of unsaturations y. IS: internal standard (n-hexatriacontane).

Analysis of lipids from ceramic sherds and foodcrusts provides a more robust approach for identifying the original contents than bulk isotope analysis. The solvent extracted residues (TLEs) from the Tianluoshan sherds analysed by gas chromatography mass spectrometry (GC-MS) revealed a range of that are not usually encountered in organic residue analysis. A range of furanose and pyranoses sugars with fragment ions at *m/z* 217 were identified as their TMS derivatives^32^. One of these, the anhydrosugar levoglucosan (1,6-anhydro-b-D-glucopyranose; *m/z* **73**, 204, 217, 147, 333) as its TMS derivative was identifiable as a major peak in five out of 20 ceramic samples and 19 out of 36 foodcrusts (Table 1, Fig. 3). Levoglucosan is a common pyrolysis product of cellulose or starch but is not usually present at such abundance in pottery vessels. It has been previously identified in charred plant material in archaeological contexts^19^ and therefore it is consistent with the use of the vessels for processing starchy materials. The compound was also easily formed by the experimental heating of rice grains and was readily absorbed into the walls of replica pottery used to cook rice at temperatures in excess of 230 °C (Fig. S1).

**Table 1:** Summary of lipid residue analysis of ceramic sherds from the Tianluoshan site.

Sample Type	Sample No.	Levoglucosan	APAA (C_18_)	APAA (C_20_)	β-sitosterol	24-Methylenecycloartanol	Campesterol	Cycloartenol	γ-tocopherol	Stigmasterol
Ceramic powder	20	5 (25%)	3 (15%)	1 (5%)	6 (30%)	1 (5%)	6 (30%)	0 (0%)	0 (0%)	4 (20%)
Foodcrusts	36	19 (53%)	25 (69%)	3 (8%)	18 (50%)	8 (22%)	10 (28%)	6 (17%)	4 (11%)	10 (28%)
Total	56	24 (43%)	28 (50%)	4 (7%)	24 (43%)	9 (16%)	16 (29%)	6 (11%)	4 (7%)	14 (25%)

In addition, a range of plant phytosterols, such as 24-methylene cycloartanol, campesterol, stigmasterol, β-sitosterol and cycloartanol^33,34^ were frequently identified in the archaeological samples, especially in the foodcrusts (Table 1). Other plant derived phenolic compounds such as γ-tocopherol (*m/z* **502**, 237, 73), notably a major component of rice oil^33^, were identified in four of the foodcrusts (Table 1). Another major class of compounds observed in the acidified methanol extracts (AE) were ω-(o-alkylphenyl)alkanoic acids (APAAs)^35^. Isomers of the APAA with 18 carbon atoms (ω-(o-alkylphenyl)octadecanoic acid) were identified in 25 out of 36 foodcrusts and three out of 20 ceramic powders (Table 1). These are formed from heating of mono- and polyunsaturated fatty acid precursors (C_18:x_) present in a range animal fats, plant and aquatic oils^36^. Interestingly, a similar distribution of C_18_ APAA isomers (dominance of the isomers where n = 3 and n = 4; Fig. 3B) were readily formed experimentally by boiling rice and charring of rice grains in pottery (Table S4, Fig. S2), where as a broader distribution of isomers is reported in marine and freshwater fats and oils^36^.

Additional products were also identified in the pottery. In three samples (TLS01, TLS1028F, TLS1033F) C_20_ and C_22_ APAAs were present in addition to the C_18_ isomers. These are derived from heating C_20:x_ and C_22:x_ fatty acid precursors that are much less abundant in terrestrial organisms and were found with at least one isoprenoid fatty acid and meet the criteria for aquatic oil identification^36^. Also, the ratio of diastereomers of phytanic acid present in five absorbed residues and six foodcrusts were calculated to identify the contribution from ruminant animals and/or aquatic organisms using established criteria^37^. Although these Tianluoshan samples show a lower SRR% (mean 72.3%, median 72.0%) than the Korean Neolithic pottery with a clear marine residue (mean 76.9%, median 81.2%)^24^, some input from aquatic oils to Tianluoshan pottery cannot be ruled out. Finally, lipids extracted from one vessel (TLS1015F) were indicative beeswax^38^, including palmitic wax esters with 40–50 carbon atoms, odd-numbered alkanes with 25–29 carbon atoms and even numbered alkanols with 24–32 carbon atoms (Fig. 3C), marking the first secure identification of this product in East Asian pottery. Applying lipid residue analysis to East Asian pottery more widely needed to fill the gap of use of beeswax and honeybee, both geographically and chronologically.

### Gas chromatography combustion isotope ratio mass spectrometry (GC-c-IRMS)

**Figure 4:**
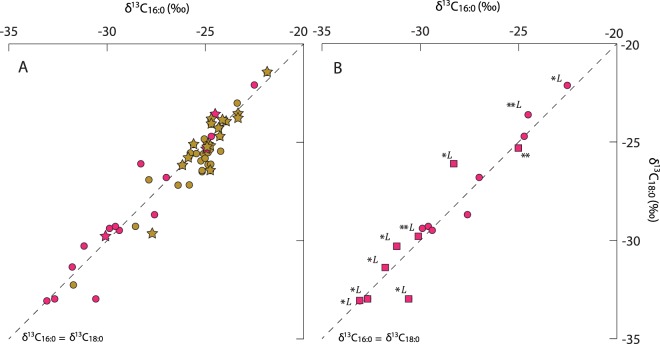
(**A**) Plot of the δ^13^C values of C_16:0_ and C_18:0_ *n*-alkanoic acids extracted from potsherds and foodcrusts from Tianluoshan, Yangtze (red) and Sejuk, Korean peninsula (yellow)^24^. Star symbols represent samples containing aquatic derived compounds interpreted from APAA(C_20,22_) with at least one isoprenoid fatty acids^35,36^. (**B**) Plot of the δ^13^C values of C_16:0_ and C_18:0_ *n*-alkanoic acids extracted from potsherds (circle) and foodcrusts (square) from Tianluoshan. Samples with levoglucosan are marked with L, while that with APAA C18 are an asterisk and APAA C18-22 are two asterisks, respectively.

Stable carbon isotope values (δ^13^C) of two saturated fatty acids (C_16:0_ and C_18:0_) were measured to provide further information on the origin of the residues. The results show wide distribution ca. −33‰ to −22‰ for both compounds (Fig. 4). As there are no C_4_ plant crops found at this site, the compounds enriched in ^13^C are attributed to lipids derived from marine organisms, presumably fish or marine mammals from the East Sea coast. However, the majority of the Tianluoshan samples analysed had relatively depleted δ^13^C values consistent C3 plants, terrestrial or freshwater fish which cannot be further distinguished using this approach alone^23^. Several of these also contained starch/cellulose pyrolysis products and other plant derived compounds, although similar lipids were also found in vessels with enriched δ^13^C (Fig. 4, Tables S2 and S3) implying that plants and fish were processed either together or sequentially. Compared to pottery from the contemporary Sejuk, shell midden in Korea, the Tianluoshan vessels have more depleted lipid ^13^C values indicating a greater reliance on terrestrial, or possibly freshwater resources. Interestingly the values of foodcrusts (−33.1‰ to −22.5‰ for C_16:0_, −33.1‰ to −22.1‰ for C_18:0_) show much wider range than that of ceramic powder (−29.9‰ to 24.5‰ for C_16:0_, −29.4‰ to −23.6‰ for C_18:0_) perhaps as the ceramic powders containing lipid accumulated over a longer-period resulting in a more attenuated signal.

## Discussion

The Tianluoshan pottery revealed a complex distribution of lipids not commonly encountered in organic residue analysis and markedly different from pottery residues from other East Asian early Holocene sites so far investigated^23–25,29^. These are characterised by lower δ^15^N values, the presence of sugars, including the degradation products of starch/cellulose, a relatively high abundance of plant sterols and APAAs derived from polyunsaturated fatty acids. Together, the molecular evidence shows that plant foods were cooked/heated in pottery during the Early Neolithic at this site given that the compounds identified are thermal degradation products of more labile precursors.

As Tianluoshan has been closely linked with early rice domestication^39^, it is tempting to interpret the organic residue data as evidence of processing this important commodity. If so, our findings would imply that rice was common culinary commodity used in utilitarian cooking pots shortly after its domestication. Certainly, the organic data are consistent with degraded rice as a major component of many of the residues, however it is important to note the presence of other wild starchy plants present in the archaeobotanical assemblage, such as acorns, foxnuts and water chestnuts. These wild foods cannot be ruled out, particularly as these were important foodstuffs in their own right and are even considered to be ‘cultivated’ at this time^40^. Acorns in particular seem to be one of the staple foods at Tianluoshan especially in the earlier phases of the site. Grinding stones were largely absent^41^ so boiling nuts and acorns in ceramic vessels to remove toxic tannic and gallic acids would have offered an alternative strategy to render them edible.

Fishing also was an important subsistence activity at Tianluoshan, shown by the recovery of large quantities of freshwater fish bone^42^ as well as marine fish, such as tuna, shark, grouper and whale^43^, yet the evidence for processing fish in pottery was very limited as shown in Table 1 with the low occurrence of sample with APAA(C_20_). Although the δ^13^C values of many of the Tianluoshan samples are as depleted as freshwater resources reference material^23^, the identification of levoglucosan confirms they are more likely derived from plant resources. As with other East Asian sites, pottery at Tianluoshan viewed as an ‘all purpose’ artefact but rather had a restricted range of uses. Importantly, however, the culinary preference at this site was for processing starchy plant foods in pottery whereas at coeval sites in Japan and Korea pottery use was strongly orientated towards aquatic products^22–25^, with little evidence for plant derived compounds despite extensive analysis. The fact that rice cultivation was beginning to be practiced in China at this time whereas contemporary sites in Japan and Korea were occupied by hunter-gatherers may explain this difference although secure identification of rice in the Tianluoshan ceramics is needed to confirm, and the difference might simply be related to separate culinary traditions.

The pattern observed at Tianluoshan also contrasts sharply with pottery use by Western Eurasian early farmers where ruminant dairy and meat products tend to dominate^9,44,45^. In Western Eurasia pottery spread along with domesticated animals and plants making a clear ‘Neolithic’ horizon whereas in China pottery and farming followed separate trajectories. There is a long history of pottery use by hunter-gatherers with the earliest vessels dating back to 18 ka cal BC in the South China^46^ long before plant and animal domestication^21^. The tradition of processing of starchy plants in pottery, including varieties of wild rice, might date back to ceramic producing hunter-gatherers of this region, in-keeping with the long-term process of domestication of this crop^39,47^. Of the starchy foods available, why rice came to become such an important staple regionally and subsequently the world’s most important food crop is a key question and still the subject of much debate among archaeobotanists and geneticists^48^. Further molecular work to securely identify rice in pottery, perhaps using protein-based approaches, could greatly contribute to this question by providing knowledge of the cultural significance and culinary uses of the crop during the early stages of its domestication and in relation to other wild foods. A residue approach would also allow the dispersal of the crop to be traced through regions where there is an absence of archaeobotanical evidence. The data presented here provide the first evidence of pottery use at an early rice domestication site and, while it was not possible to identify this product securely, they should at the very least provide a useful comparator for further studies.

## Material and Methods

### Heating/Cooking Experiment

Experiments were carried out to determine whether organic residues are formed through rice processing, and if so the types of compounds that are formed. Firstly, white and brown Japonica rice were cooked in field fired replica ceramic vessels for ca. 1 h. The ceramics were emptied following the experiments and air dried and wrapped in foil for further analysis. Samples of the rice grains used in the cooking experiments were also heated in foil (230 °C, 270 °C and 310 °C) for 15 min and compared with uncharred controls to examine thermal alteration to any detectable compounds.

### Elemental Analysis Isotope Ratio Mass Spectrometry (EA-IRMS)

Foodcrusts (~2 mg) were sampled from archaeological (n = 36) and experimental (n = 5) vessels with a clean scalpel and were crushed to homogenise using a clean agate pestle and mortar. These were directly analysed by elemental analysis isotope ratio mass spectrometry (EA-IRMS) to determine their bulk stable carbon (δ^13^C) and nitrogen isotope (δ^15^N) values, as already described^49^. Samples yielding less than 1% N were eliminated from objectives for interpretation. Instrument precision on repeated measurements was ±0.2‰ (s.e.m.). δ^13^C, δ^15^N = [(Rsample/Rstandard−1)] × 1,000, where R = ^13^C/^12^C and ^15^N/^14^N. All sample measurements are expressed in per mil relative to the standard for δ^13^C is Vienna PeeDee Belemnite (V-PDB) and the standard for δ^15^N is air N_2_, respectively.

### Lipid residue analysis

Lipids were extracted from archaeological (20 ceramic powder and 36 foodcrusts) and experimental samples (8 ceramic powder and 5 foodcrusts) using two kinds of extraction method by following the established methods, the solvent extraction^50^ and acid extraction^22^. Extracted lipids were analyzed by GC-MS (Gas Chromatography-Mass Spectrometry), HT-GC-FID-MS (High temperature- GC- Flame ionization detector -MS) and GC-c-IRMS (Gas Chromatography-combustion-Isotope Ratio Mass Spectrometry).

For GC-MS, an Agilent 7890A series chromatograph attached to an Agilent 5975C Inert XL mass-selective detector with a quadrupole mass analyser (Agilent technologies, Cheadle, Chershire, UK) was used. A splitless injector was used and kept at 300 °C. For scanning, the GC column (DB-5ms (5%-phenyl)-methylpolysiloxane column, 30 m × 0.250 mm × 0.25 µm; J&W Scientific, Folsom, CA, USA) was inserted into the ion source directly. Helium was used as the carrier gas, keeping the flow at a rate of 3 mL min^−1^. The ionisation energy of the MS was 70 eV and spectra were obtained between *m/z* 50 and 800. For further detection of phytanic acid diastereomers, a selected ion monitoring (SIM) method was used with a DB23 (50%-Cyanopropyl)-methylpolysiloxane column (60 m × 0.250 mm × 0.25 µm; J&W Scientific, Folsom, CA, USA) following the previous studies by the authors^24,37^.

For HT-GC-FID-MS, derivatized extracts (1 *μ*L) were also analysed with a cold on-column injector inserted into a DB5-HT column (15 m × 0.32 mm × 0.1 *μ*m; Agilent, UK) using an Agilent GC with the column effluent split (50:50) between an Agilent 5975 C Inert XL mass-selective detector and an Agilent flame ionization detector (FID). The oven temperature was set at 50 °C for 1 min, then raised by 15 °C/min to 100 °C, then 10 °C/min to 375 °C and held for 10 mins. The inlet temperature tracked the oven temperature program. The FID was set at a temperature of 350 °C. Helium was used as a carrier gas.

For GC-c-IRMS, an Isoprime 100 (Isoprime, Cheadle, UK) linked to a Hewlett Packard 7890B series GC (Agilent Technologies, Santa Clara, CA, USA) with an Isoprime GC5 interface (Isoprime Cheadle, UK) was used. One microlitre of each sample was injected into DB-5MS ultra-inert fused-silica column. Eluted products were ionized in the mass spectrometer by electron impact and ion intensities of *m/z* 44, 45 and 46 were recorded for automatic computing of the ^13^C/^12^C ratio of each peak in the extracts. Computation was made with IonVantage and IonOS softwares (Isoprime, Cheadle, UK) and was based on comparisons with standard reference gas (CO_2_) of known isotopic composition that was repeatedly measured. The results of the analysis were expressed in per mill (‰) relative to an international standard, V-PDB. The accuracy and precision of the instrument was determined on n-alkanoic acid ester standards of known isotopic composition (Indiana standard F8-3). Each sample was measured in replicate (mean of S.D. 0.11‰ for C16:0 and 0.10‰ for C18:0). Values were also corrected subsequent to analysis to account for the methylation of the carboxyl group that occurs during acid extraction.

## Electronic supplementary material

Table S1, Table S2, Table S3, Table S4, Figure S1, Figure S2

## Data Availability

All data related to this article which is summarised in the submitted supporting information files is available online at ADS archive arch-3269-1 by Archaeology Data Service, University of York (https://doi.org/10.5284/1048322).
