# Supplementary material for: Molecular and isotopic evidence for the processing of starchy plants in Early Neolithic pottery from China
Source: Sci Rep. 2018 Nov 19;8:17044. doi: 10.1038/s41598-018-35227-4 (PMC6242940; doi:10.1038/s41598-018-35227-4)
Supplement: Supplementary file 1 — Table S1, Table S2, Table S3, Table S4, Figure S1, Figure S2 [file 41598_2018_35227_MOESM1_ESM.docx]

# **Molecular and isotopic evidence for the processing of starchy plants in Early Neolithic pottery from China**

**Supplementary dataset**

Shinya Shoda^a, b^, Alexandre Lucquin^a^, Chi Ian Sou^a^, Yastami Nishida^c^, Guoping Sun^d^, Hiroshi Kitano^e^, Joon-ho Son^f^, Shinichi Nakamura^g^ and Oliver E. Craig^a^

a BioArCh, University of York / Wentworth Way, Heslington, York, YO10 5NG, UK

b Nara National Research Institute for Cultural Properties / Nijo 2-9-1, Nara, Nara, 630-8577, Japan

c Niigata Prefectural Museum of History / Sekihara 1, Nagaoka, Niigata, 940-2035, Japan

d Zhejiang Provincial Institute of Relics and Archaeology / 26 Jiashan Xincun, Juashan Road, Hangzhou, Zhejiang, 310014, China

e Tohoku University of Art and Design / Kamisakurada 3-4-5, Yamagata, Yamagata, 990-9530, Japan

f Korea University / 2511 Sechong-ro, Jochiweon-up, Sejong-si, 339-700, South Korea

g Kanazawa University / Kakuma, Kanazawa, Ishikawa, Ishikawa, 920-1192, Japan

Corresponding author: Shinya Shoda shinya.shoda@york.ac.uk

***Table S1. The AMS dating for the charred deposits adhering to the pottery vessel wall (foodcrust) from the Tianluoshan site and their calibrated ages. Calibration was performed using Oxcal v.4.2.4 (Bronk Ramsely 2013), with atmospheric data by Reimer et al. (2013).***

| Measurement ID | Sample ID | Layer | Material | δ^13^C (‰) | ^14^C age BP (yrBP±1σ) | Calibrated Age (2σ) |
| --- | --- | --- | --- | --- | --- | --- |
|  |  |  |  |  |  |  |
| PED-32364 | TLS1008F | 8 | foodcrust | -29.13±0.15 | 5920±25 | 4841-4724 cal BC (95.4%) |
| PED-32365 | TLS1019F | 8 | foodcrust | -26.49±0.17 | 6045±25 | 5016-4878 cal BC (87.6%) 4871-4848 cal BC ( 7.8%) |
| PED-32366 | TLS1020F | 8 | foodcrust | -25.66±0.17 | 6170±25 | 5214-5049 cal BC (95.4%) |
| PLD-34610 | TLS1015F | 8 | foodcrust | -31.41±0.28 | 5890±25 | 4827-4815 cal BC ( 3.1%) 4804-4712 cal BC (92.3%) |
| PED-35034 | TLS_WC1 | 8 | charred fruit  (water chestnut) | -32.66±0.27 | 5865±20 | 4791-4695 cal BC (95.4%) |
| PED-32371 | TLS1035F | 7 | foodcrust | -26.30±0.16 | 5920±25 | 4935-4791 cal BC (95.4%) |
| PED-32369 | TLS1028F | 6 | foodcrust | -27.48±0.16 | 5970±25 | 4935-4791 cal BC (95.4%) |
| PED-32370 | TLS1031F | 6 | foodcrust | -26.48±0.26 | 5960±25 | 4935-4777 cal BC (95.4%) |
| PED-35033 | TLS_R1 | 6 | charred fruit  (rice) | -27.98±0.23 | 5845±25 | 4788-4668 cal BC (89.4%)  4660-4656 cal BC ( 0.6%)  4638-4617 cal BC ( 5.4%) |
| PED-32367 | TLS1022F | 4 | foodcrust | -28.22±0.19 | 5890±25 | 4827-4816 cal BC ( 3.1%) 4803-4711 cal BC (92.3%) |
| PED-32368 | TLS1023F | 4 | foodcrust | -26.73±0.15 | 5980±25 | 4936-4796 cal BC (95.4%) |

Bronk Ramsey, C. 2009. Bayesian analysis of radiocarbon dates. *Radiocarbon*, 51(1), 337-360.

Reimer, P. J., Bard, E., Bayliss, A., Beck, J. W., Blackwell, P. G., Bronk Ramsey, C., Grootes, P. M., Guilderson, T. P., Haflidason, H., Hajdas, I., HattŽ, C., Heaton, T. J., Hoffmann, D. L., Hogg, A. G., Hughen, K. A., Kaiser, K. F., Kromer, B., Manning, S. W., Niu, M., Reimer, R. W., Richards, D. A., Scott, E. M., Southon, J. R., Staff, R. A., Turney, C. S. M., & van der Plicht, J. 2013. IntCal13 and Marine13 Radiocarbon Age Calibration Curves 0-50,000 Years cal BP. *Radiocarbon*, 55(4).

***Table S2. Charred deposits (foodcrusts) from the Tianluoshan site selected for bulk isotope and lipid analysis.*** *FA (Cx:y) = fatty acids with carbon length x and number of unsaturations y, Phy(x) = phytanic acid with SRR ratio as x (Lucquin et al. 2016), APAA (Cn) = ω-(o-alkylphenyl) alkanoic acids with carbon length n. tr = trace. DC (Cx) = α,ω-dicarboxylic acids with carbon length x. Lev = Levoglucosan, Sit* = *β-sitosterol, Met = 24-Methylenecycloartanol, Cam = Campesterol, Cyc = Cycloartenol, Toc = γ-tocopherol, Sti = Stigmasterol, Cho = Cholesterol, WE (C_x_) = wax esters with carbon length x, OH (C_x_) = Alkanol with carbon length x, ALK (C_x_) = Alkane with carbon length x. Radiocarbon dates associated with the vessels selected for residue analysis are also shown. Plant oils are interpreted from the presence of levoglucosan (Simoneit et al. 1999) and APAA (C_18_) without APAA (C_20, 22_) while aquatic oils are interpreted from APAA (C_20, 22_) with at least one isoprenoid fatty acids (Evershed et al. 2008). Beeswax is interpreted from the presence of hexadecanoic wax esters, even numbered alkanols and odd numbered alkanes (Heron et al. 1994).*

| **Laboratory Code** | **%C** | **δ^13^ C (‰)** | **%N** | **δ^15^ N**  **(‰)** | **C:N (atom)** | **Lipid conc. (µg g^-1^)** | **Major compound detected in solvent extracts (TLE)** | **Other major compound detected in acid extracts (AE)** | **C_16:0_ δ^13^C (‰)** | | **C_18:0_ δ^13^C (‰)** | **Interpretation** |
| --- | --- | --- | --- | --- | --- | --- | --- | --- | --- | --- | --- | --- |
| TLS01F | 47.1 | -25.0 | 1.8 | 12.6 | 30.8 | 596 | FA(C_12:0-22:0,_ C_18:1_), Lev, Sit, Cho | FA(C_17br_), DC(C_8-14_), APAA(C_16tr, 18tr_) | -22.5 | | -22.1 | plant |
| TLS19F | 50.4 | -26.4 | 5.4 | 6.9 | 10.8 | 426 | FA(C_14:0-24:0,_ C_18:1_), Lev | APAA(C_18_), Phy(53) | -28.3 | | -26.1 | plant |
| TLS20F | 37.4 | -26.1 | 2.5 | 6.7 | 17.3 | 371 | FA(C_16:0-26:0,_ C_18:1_) | APAA(C_18_) |  | |  |  |
| TLS1001F | 51.3 | -25.6 | 3.7 | 4.6 | 16.3 | 646 | FA(C_12:0-28:0,_ C_18:1_), Lev, Met, | APAA(C_18_) | -33.1 | | -33.1 | plant |
| TLS1002F | 61.8 | -25.7 | 4.1 | 4.2 | 17.7 | 36 | FA(C_14:0-26:0_) |  | |  |  |  |
| TLS1003F | 47.7 | -25.1 | 6.5 | 7.9 | 8.6 | 60 | FA(C_16:0-18:0_) |  |  | |  |  |
| TLS1004F | 47.3 | -25.9 | 5.3 | 9.5 | 10.4 | 192 | FA(C_16:0-18:0_), Lev | APAA(C_18tr_) |  | |  | plant |
| TLS1005F | 39.9 | -25.1 | 3.7 | 5.7 | 12.5 | 1013 | FA(C_14:0-26:0,_ C_18:1_), Lev, Met, Sit, Cam, Cyc, Toc, Sti | APAA(C_18_) | -30.6 | | -33.0 | plant |
| TLS1006F | 53.1 | -26.1 | 2.6 | 4.8 | 23.9 | 555 | FA(C_14:0-28:0,_ C_18:1_), Lev, Sit, Cam, Cyc, Toc, Sti | APAA(C_18_) |  | |  | plant |
| TLS1007F |  |  |  |  |  | 591 | FA(C_14:0-24:0,_ C_18:1_), Lev, Met, Sit, Cam, Toc, Sti | APAA(C_18_) | -31.8 | | -31.4 | plant |
| TLS1008F | 47.1 | -26.5 | 3.4 | 5.1 | 16.1 | 663 | FA(C_16:0-28:0,_ C_18:1_), Lev, Sit, Cam, Toc, Sti | APAA(C_18_) | -32.7 | | -33.0 | plant |
| TLS1009F | 42.6 | -25.9 | 4.0 | 6.7 | 12.4 | 571 | FA(C_14:0-24:0,_ C_18:1_), Lev_tr_, Sit, Sti | APAA(C_18tr_), Phy(89) |  | |  |  |
| TLS1010F | 42.7 | -25.5 | 3.8 | 6.7 | 13.1 | 525 | FA(C_14:0-28:0,_ C_18:1_), Sit | APAA(C_18_) |  | |  |  |
| TLS1011F | 43.9 | -25.8 | 3.8 | 5.7 | 13.5 | 458 | FA(C_14:0-26:0,_ C_18:1_), Lev, Met, Sit, Cam, Cyc, Sti | APAA(C_18_) |  | |  | plant |
| TLS1012F | 44.3 | -26.5 | 2.7 | 3.7 | 18.8 | 520 | FA(C_16:0-24:0,_ C_18:1_), Lev, Met, Sit, Cam, Cyc, Sti | APAA(C_18_) |  | |  | plant |
| TLS1013F | 40.8 | -26.5 | 2.8 | 4.7 | 17.1 | 402 | FA(C_16:0-24:0,_ C_18:1_), Lev, Sit | APAA(C_18_) |  | |  | plant |
| TLS1014F | 39.9 | -24.1 | 5.2 | 7.6 | 8.9 | 67 | FA(C_16:0-18:0,_ C_18:1_), | APAA(C_18tr_) |  | |  |  |
| TLS1015F | 39.6 | -25.3 | 3.5 | 5.7 | 13.2 | 1651 | FA(C_16:0-30:0,_ C_18:1_), WE (C_40-50_), OH (C_24-34_), ALK (C_27-33_) | APAA(C_16, 18_) |  | |  | bees-wax |
| TLS1016F | 52.7 | -26.4 | 3.8 | 3.0 | 16.1 | 579 | FA(C_16:0-26:0,_ C_18:1_), Lev, Met, Sit, Cam, Cyc, Sti | APAA(C_18_) |  | |  | plant |
| TLS1017F | 50.4 | -24.6 | 3.3 | 1.6 | 18.0 | 368 | FA(C_16:0-24:0,_ C_18:1_), Lev, Sit | APAA(C_18_) |  | |  | plant |
| TLS1018F | 42.6 | -25.4 | 3.6 | 4.4 | 13.9 | 283 | FA(C_14:0-30:0_), Lev, Sit, Cam | APAA(C_18_) |  | |  | plant |
| TLS1022F | 40.4 | -26.1 | 2.1 | 4.7 | 22.9 | 73 | FA(C_16:0-18:0_) |  |  | |  |  |
| TLS1023F | 48.0 | -25.2 | 7.6 | 7.4 | 7.3 | 222 | FA(C_14:0-24:0,_ C_18:1_), Lev, Met, Sit, Cam, Sti | FA(C_17br_), APAA(C_18_), Phy | | |  | plant |
| TLS1024F | 32.8 | -25.7 | 2.6 | 7.9 | 14.9 | 50 | FA(C_16:0-18:0_) |  |  | |  |  |
| TLS1025F | 23.0 | -25.9 | 1.6 | 4.5 | 17.2 | 81 | FA(C_16:0-18:0tr_) |  |  | |  |  |
| TLS1026F | 27.0 | -25.3 | 2.0 | 6.2 | 15.8 | 88 | FA(C_16:0-18:0_) |  |  | |  |  |
| TLS1027F | 41.4 | -26.3 | 3.0 | 6.3 | 16.2 | 57 | FA(C_16:0-18:0tr_) |  |  | |  |  |
| TLS1028F | 42.0 | -25.7 | 5.1 | 9.8 | 9.6 | 525 | FA(C_14:0-28:0,_ C_18:1,_ C_17br_), APAA(C_18, 20, 22tr_), Phy, Lev, Sit, Cam | FA(C_17br_), APAA(C_18, 20, 22tr_), Phy(82) | -30.1 | | -29.8 | plant/ aquatic |
| TLS1029F | 41.9 | -26.0 | 3.7 | 7.3 | 13.4 | 275 | FA(C_16:0-18:0,_ C_18:1_), Lev, Sit |  |  | |  | plant |
| TLS1030F | 48.2 | -25.6 | 3.7 | 7.9 | 15.3 | 122 | FA(C_16:0-24:0_) | APAA(C_18, 20tr_) |  | |  |  |
| TLS1031F | 40.2 | -24.5 | 4.2 | 7.9 | 11.1 | 243 | FA(C_14:0-30:0,_ C_18:1,_), Sit | FA(C_15,17br_), APAA(C_18, 20tr_), Phy _tr_ |  | |  | plant/ aquatic |
| TLS1032F | 35.8 | -25.2 | 5.0 | 10.6 | 8.4 | 261 | FA(C_14:0-24:0,_ C_16:1-18:1_) | APAA(C_18_), Phy(72) |  | |  |  |
| TLS1033F | 38.4 | -23.5 | 5.0 | 9.3 | 9.0 | 451 | FA(C_14:0-18:0,_ C_18:1_) | DC(C_9-11_), APAA(C_18, 20, 22tr_), Phy(79) | -25.0 | | -25.3 |  |
| TLS1034F | 12.4 | -26.7 | 0.7 | 8.1 | 19.5 | 75 | FA(C_16:0-18:0_) |  |  | |  |  |
| TLS1035F |  |  |  |  |  | 280 | FA(C_16:0-26:0,_ C_18:1_), Lev, Met, Sit, Cam, Cyc, Sti | APAA(C_18_), Phy(85) | -31.2 | | -30.3 | plant |
| TLS1036F | 42.3 | -25.0 | 3.9 | 10.3 | 12.8 | 110 | FA(C_16:0-24:0_) | APAA(C_18_) |  | |  |  |

Evershed, R. P., M. S. Copley, L. Dickson, and F. A. Hansel. 2008. Experimental Evidence For The Processing Of Marine Animal Products And Other Commodities Containing Polyunsaturated Fatty Acids In Pottery Vessels. *Archaeometry* 50 (1). Blackwell Publishing Ltd: 101–13.

Heron, C., N. Nemcek, and K. M. Bonfield. 1994. The Chemistry of Neolithic Beeswax. *Naturwissenschaften* 81(6): 266-269.

Lucquin A, Colonese AC, Farrell TFG, Craig OE. 2016 Utilising phytanic acid diastereomers for the characterisation of archaeological lipid residues in pottery samples. *Tetrahedron Lett*. 57, 703–707.

Simoneit, B. R. T., J. J. Schauer, C. G. Nolte, D. R. Oros, V. O. Elias, M. P. Fraser, W. F. Rogge, and G. R. Cass. 1999. Levoglucosan, a Tracer for Cellulose in Biomass Burning and Atmospheric Particles. *Atmospheric Environment* 33 (2): 173–82.

***Table S3. Pottery sherds from the Tianluoshan site selected for lipid analysis (extracted by acid extraction).*** *FA (Cx:y) = fatty acids with carbon length x and number of unsaturations y, Phy(x) = phytanic acid with SRR ratio as x (Lucquin et al. 2016), APAA (Cn) = ω-(o-alkylphenyl) alkanoic acids with carbon length n. tr = trace. DCx = α,ω-dicarboxylic acids with carbon length x. Lev = Levoglucosan, Sit* = *β-sitosterol, Met = 24-Methylenechcloartanol, Cam = Campesterol, Cyc = Cycloartenol, Toc = γ-tocopherol, Sti = Stigmasterol, Cho = Cholesterol. Plant oils are interpreted from the presence of levoglucosan (Simoneit et al. 1999) and APAA (C_18_) without APAA (C_20, 22_) while aquatic oils are interpreted from APAA (C_20, 22_) with at least one isoprenoid fatty acids (Evershed et al. 2008).*

| **Laboratory Code** | **Lipid conc. (µg g^-1^ )** | **Major compound detected** | **C_16:0_ δ^13^C (‰)** | **C_18:0_ δ^13^C (‰)** | **Interpretation** |
| --- | --- | --- | --- | --- | --- |
| TLS01 | 62 | FA(C_11:0-24:0,_ C_16:1-18:1_), DC(C_8-12_), APAA(C_18, 20, 22tr_), Phy(66), Lev, Met, Sit, Cam | -24.5 | -23.6 | plant/aquatic |
| TLS02 | 2 | FA(C_16:0-18:0_) |  |  |  |
| TLS03 | 40 | FA(C_14:0-26:0,_ C_16:1-18:1_), DC(C_9-11_) | -29.4 | -29.5 |  |
| TLS04 | 3 | FA(C_16:0-24:0,_ C_16:1-18:1_), Phy(tr) |  |  |  |
| TLS05 | 4 | FA(C_16:0-18:0,_ C_18:1_), Lev, Sit, Cam, Sti |  |  |  |
| TLS06 | 3 | FA(C_16:0-18:0,_ C_18:1_), Lev, Sit, Cam, Sti |  |  |  |
| TLS07 | 2 | FA(C_16:0-18:0,_ C_18:1_), Lev, Sit, Cam, Sti |  |  |  |
| TLS08 | 3 | FA(C_16:0-18:0,_ C_18:1_), Lev, Sit, Cam, Sti |  |  |  |
| TLS09 | 14 | FA(C_14:0-30:0,_ C_16:1-18:1_), Sit, Cam |  |  |  |
| TLS10 | 3 | FA(C_16:0-18:0_) |  |  |  |
| TLS11 | 101 | FA(C_12:0-26:0,_ C_16:1-18:1_), DC(C_9-13_) | -24.7 | -24.7 |  |
| TLS12 | 10 | FA(C_14:0-28:0,_ C_18:1_) | -29.9 | -29.4 |  |
| TLS13 | 13 | FA(C_14:0-26:0,_ C_18:1_) | -29.6 | -29.3 |  |
| TLS14 | 10 | FA(C_10:0-24:0,_ C_16:1-18:1,_ C_15br, 17br_ ), DC(C_9-12_), APAA(C_18_), Phy(82) |  |  |  |
| TLS15 | 6 | FA(C_14:0-24:0,_ C_16:1-18:1,_ C_15br, 17br_) |  |  |  |
| TLS16 | 139 | FA(C_14:0-26:0,_ C_16:1-18:1_) | -27.0 | -26.8 |  |
| TLS17 | 6 | FA(C_12:0-18:0,_ C_16:1_) |  |  |  |
| TLS18 | 20 | FA(C_14:0-24:0,_ C_16:1-18:1,_ C_17br_ ), DC(C_9-11_), Phy(56) | -27.6 | -28.7 |  |
| TLS19 | 8 | FA(C_14:0-24:0,_ C_16:1-18:1,_ C_15br, 17br_ ), APAA(C_18_), Phy(65) |  |  |  |
| TLS20 | 6 | FA(C_12:0-24:0,_ C_16:1-18:1_), DC(C_9-12_), APAA(C_16, 18_), phy(67) |  |  |  |

Evershed, R. P., M. S. Copley, L. Dickson, and F. A. Hansel. 2008. Experimental Evidence For The Processing Of Marine Animal Products And Other Commodities Containing Polyunsaturated Fatty Acids In Pottery Vessels. *Archaeometry* 50 (1). Blackwell Publishing Ltd: 101–13.

Lucquin A, Colonese AC, Farrell TFG, Craig OE. 2016. Utilising phytanic acid diastereomers for the characterisation of archaeological lipid residues in pottery samples. *Tetrahedron Lett*. 57, 703–707.

Simoneit, B. R. T., J. J. Schauer, C. G. Nolte, D. R. Oros, V. O. Elias, M. P. Fraser, W. F. Rogge, and G. R. Cass. 1999. Levoglucosan, a Tracer for Cellulose in Biomass Burning and Atmospheric Particles. *Atmospheric Environment* 33 (2): 173–82.

***Table S4. ω-(o-alkylphenyl)*** ***alkanoic acid with carbon length 18 (APAA (C_18_)) and Levoglucosan detected from experimental pottery sherds, foodcrusts and rice grains.***

| **Lab Code** | **Sample type** | **Lipid conc. (mg g^-1^)** | **Levoglucosan** | **APAA(C_18_)** |
| --- | --- | --- | --- | --- |
| RiEx11TLE | pottery sherd | 0.0 | Y | n/a |
| RiEx11FTLE | foodcrust | 0.4 | - | n/a |
| RiEx12TLE | pottery sherd | 0.0 | - | n/a |
| RiEx41TLE | pottery sherd | 0.0 | Y | n/a |
| RiEx41FTLE | foodcrust | 3.4 | Y | n/a |
| RiEx42TLE | pottery sherd | 0.0 | Y | n/a |
| MoBRi0TLE | brown rice grains non-heated | 13.0 | - | n/a |
| MoBRi230TLE | brown rice grains heated to 230 °C | 2.8 | Y | n/a |
| MoBRi270TLE | brown rice grains heated to 270 °C | 8.6 | Y | n/a |
| MoBRi310TLE | brown rice grains heated to 300 °C | 4.5 | Y | n/a |
| RiEx11AE | pottery sherd | 0.0 | n/a | - |
| RiEx11FAE | foodcrust | 4.4 | n/a | Y |
| RiEx12AE | pottery sherd | 0.0 | n/a | - |
| RiEx41AE | pottery sherd | 0.0 | n/a | - |
| RiEx41FAE | foodcrust | 10.3 | n/a | Y |
| RiEx42AE | pottery sherd | 0.0 | n/a | - |
| MoBRi0AE | brown rice grains non-heated | 13.1 | n/a | - |
| MoBRi230AE | brown rice grains heated to 230 °C | 12.5 | n/a | Y |
| MoBRi270AE | brown rice grains heated to 270 °C | 10.5 | n/a | Y |
| MoBRi310AE | brown rice grains heated to 300 °C | 6.8 | n/a | Y |

**Figure S1:** Partial chromatogram by TLE (total lipid extraction or solvent extraction) samples from experimental rice charring. A - non-heated (MoBRi0TLE), B - heated at 230°C for 30 minutes (MoBRi230TLE), C - heated at 270°C for 30 minutes (MoBRi270TLE), D - heated at 310°C for 30 minutes (MoBRi310TLE). This shows how increasing temperature of cooking increases the peaks of Levoglucosan. IS: internal standard (n-hexatriacontane).

**Figure S2:** Mass chromatogram (*m/z* 290) showing isomeric distribution C_18_ APAAs extracted with acidified methanol from an experimental rice charring heated at 270°C for 30 minutes (MoBRi270AE) resolved on a DB-23 column in SIM mode.
